# Patient perspectives on continuous glucose monitoring system (CGMS) for diabetes in Malaysia: a vital voice in health technology assessment (HTA) informing decision-making

**DOI:** 10.1017/S0266462325000078

**Published:** 2025-02-06

**Authors:** Nur Farhana Mohamad, Ana Fizalinda Abdullah Sani, Nurfarah Aqilah Ahmad Nizam, Foo Sze-Shir, Izzuna Mudla Mohamed Ghazali, Roza Sarimin

**Affiliations:** Malaysian Health Technology Assessment Section (MaHTAS), Medical Development Division, Ministry of Health Malaysia

**Keywords:** Health Technology Assessment (HTA), Continuous Glucose Monitoring Systems (CGMS), diabetes patients, patients’ perspective, decision-making

## Abstract

**Objective:**

To integrate patient perspectives into Health Technology Assessment (HTA) by exploring the perceived benefits, barriers, and expectations of diabetes patients and their caregivers in Malaysia regarding the use of CGMS.

**Methods:**

This qualitative study employed focus group discussions (FGDs) with 30 participants, including adults, adolescents, and caregivers managing insulin-requiring diabetes, conducted between May and September 2023 in Kuala Lumpur and Putrajaya, Malaysia. Participants were recruited through purposive sampling. Data were collected using semi-structured interviews and analyzed thematically to capture insights into CGMS benefits, barriers, and expectations.

**Results:**

Participants highlighted CGMS as a transformative tool, offering real-time data, improving glycemic control, and enhancing quality of life by reducing anxiety and the burden of frequent glucose checks. Despite these benefits, significant barriers were identified, including high costs, limited access, technical issues, and social stigma, particularly among adolescents. There was a strong call for government subsidies, better technical support, and healthcare provider training to optimize CGMS use. Patient perspectives were integrated into the HTA alongside systematic reviews and economic evaluations, directly informing policy recommendations, including prioritizing CGMS for high-risk T1DM patients and exploring subsidy frameworks to improve affordability.

**Conclusions:**

Patient perspectives serve as a vital voice in HTA, offering real-world insights that enhance the understanding of CGMS in diabetes management. Addressing financial, technical, and social barriers is crucial to improving CGMS accessibility and usability. By embedding patient perspectives into HTA and strengthening partnerships across healthcare systems, patient perspectives become instrumental in shaping patient-centered policies and informing equitable decision-making on CGMS utilization in Malaysia.

## Introduction

Diabetes mellitus is a chronic condition with significant impacts on individuals and caregivers (1). It remains a major public health issue in Malaysia, contributing to increased complications, disabilities, and mortality, and imposing a significant economic burden, with an estimated annual cost of USD 600 million (2;3). In the pursuit of enhancing diabetes management, the Continuous Glucose Monitoring System (CGMS) has emerged as a technological innovation, offering real-time insights into glucose levels and potentially transforming the landscape of diabetes care. Over the last few years, CGMS has proven to be a paradigm shift in both the management of diabetes and the understanding of the disease (4).

Continuous Glucose Monitoring System (CGMS) continuously measures glucose levels, providing insights into blood glucose fluctuations (5). Real-time and intermittently scanned CGMS has enabled diabetes patients, including children and adolescents with Type 1 Diabetes Mellitus (T1DM), to adjust diet, activity, and treatment, improving glycemic control, reducing complications, and making intensive treatment safer by preventing hypoglycemia (5;6). In Malaysia, CGMS has been used in research at selected clinical centers, but its use remains limited and self-funded by a small group of patients.

Recognizing the potential of CGMS to improve diabetes care in Malaysia, the Ministry of Health’s Malaysian Health Technology Assessment Section (MaHTAS) conducted a Health Technology Assessment (HTA) to evaluate CGMS in terms of effectiveness, safety, and cost-effectiveness, as well as organizational and psychosocial aspects. Traditionally, HTAs focused on quantitative assessments, often overlooking social, ethical, and political dimensions (7). However, patient and public involvement (PPI) has increasingly become part of the HTA process, enriching assessments with patients’ firsthand insights into living with specific conditions and the impact of healthcare technologies (7–9).

Existing evidence shows that CGMS benefits various groups, including children, adolescents, and adults with T1DM and some with Type 2 Diabetes Mellitus (T2DM) (6). However, concerns remain about the overwhelming data and potential disruptions from false alarms, which can impact patients’ quality of life (7). There is also a lack of comprehensive patient perspectives on CGMS use in Malaysia. In line with global efforts to involve patients in HTA, (10) this qualitative study aims to gather feedback and experiences on CGMS use among insulin-requiring diabetes patients. These insights will assist decision-makers in evaluating the suitability and effectiveness of CGMS in local settings within the HTA framework.

## Methods

The study aimed to capture the experiences of diabetes patients using CGMS, focusing on their views regarding its benefits, barriers, challenges, and expectations. Conducted from May to September 2023 in Kuala Lumpur and Putrajaya, Malaysia, the research used focus group discussions (FGDs) with adults, adolescents, and caregivers managing insulin-requiring diabetes. Participants were recruited through purposive sampling via emails, flyers, and clinician referrals. Individuals with insulin-requiring T1DM or T2DM, either adolescents aged 13–18 or adults over 18 and fluent in Malay or English, were eligible to participate. Patients included in the study predominantly used a limited range of CGMS devices available in Malaysia, with the majority utilizing the Freestyle Libre, which comprises two main components: a small sensor typically applied to the back of the upper arm and a reader device or smartphone app for scanning the sensor to access glucose data. Most patients used insulin injections to manage their diabetes, while only a few were on insulin pumps. The study team comprised medical doctors with postgraduate qualifications in public health and a research officer, all trained in qualitative research and analysis, with some prior experience in conducting qualitative studies. Data was gathered through socio-demographic forms and FGDs, using a semi-structured interview guide adapted from the European Network for Health Technology Assessment (EUnetHTA) with input from clinical experts (refer Table 1: Semi-structured interview guide for details). Each 90-minute session was moderated, recorded, transcribed, and, when necessary, translated into English. The transcripts were then analyzed using thematic analysis with Atlasti.23 software. Participation was voluntary, with anonymity and confidentiality maintained. The study was registered under the National Medical Research Register (NMRR ID-23-00721-OKL (IIR)), approved by the Medical Research & Ethics Committee, Ministry of Health, Malaysia, and adhered to the Declaration of Helsinki and Malaysian Good Clinical Practice Guideline.

**Table 1.** Semi-structured interview guide

Introduction:
Researchers introduce themselves, explain the research purpose, describe group discussion rules, ask for any queries, and take consent
**Warm-up:**
Participants described their use of CGMS Prompts: What type of CGMS you are using? How long have you used CGMS? How did you get the CGMS? How do you pay for the CGMS?
**Key questions:**
Participants’ experiences of CGMS Prompts: What are the participants’ experiences of using CGMS? What are the impacts of CGMS on their diabetes care? What are the benefits of using CGMS? What are the problems in using CGMS? Participants’ expectations for CGMS Prompts: What are the participants’ expectations for CGMS? What can be improved?

## Results

The sample consisted of 30 individuals, as detailed in Table 2: Participant Characteristics. This included 14 adult patients (29 percent Malay, 36 percent Chinese, 29 percent Indian, and 7 percent Others, aged 20 to 80). Of these, 12 were adult patients with T1DM, and 2 were adult patients with T2DM. Additionally, there were eight adolescent patients (50 percent Malay, 25 percent Chinese, and 25 percent Indian, aged 11 to 18) and eight caregivers (50 percent Malay, 25 percent Chinese, and 25 percent Indian, aged 43 to 53). Among these, most were within the low- and middle-income groups. The duration of CGMS use ranged from two weeks to eight years. The majority of patients indicated that CGMS was recommended by their endocrinologist, with most bearing the financial burden of the device independently, without external financial assistance. Four focus group discussions were conducted, each involving seven to eight participants, including adults, adolescents, and caregivers. Data saturation was achieved after the fourth group discussion.

**Table 2.** Participant characteristics (N = 30)

Category	N (%)	Mean (Sd)	Range
Adults			
Age (Years)		40.6 (18.7)	20–80
Gender	Male: 10 (71.4) Female: 4 (28.6)		
Race	Malay: 4 (28.6) Chinese: 5 (35.7) Indian: 4 (28.6) Others: 1 (7.1)		
Income Levels	Low-income: 5 (35.7) Middle-income: 8 (57.1) High-income: 1 (7.1)		
Duration of CGMS Use (Years)		1.71 (2.0)	0.04–8
Adolescents			
Age (Years)		15.5 (2.6)	11–18
Gender	Male: 4 (50.0) Female: 4 (50.0)		
Race	Malay: 4 (50.0) Chinese: 2 (25.0) Indian: 2 (25.0)		
Duration of CGMS Use (Years)		1.63 (1.7)	0.04–5.5
Caregivers			
Age (Years)		48.0 (4.2)	43–53
Gender	Male: 3 (37.5) Female: 5 (62.5)		
Race	Malay: 4 (50.0) Chinese: 2 (25.0) Indian: 2 (25.0)		
Income Levels	Low-income: 4 (50.0) Middle-income: 2 (25.0) High-income: 2 (25.0)		

The focus group discussions revealed a few emerging themes: perceived benefits of CGMS, perceived barriers to CGMS, and expectations for CGMS adoption. The detailed patient transcripts supporting these themes are provided in the appendix for reference.

### Perceived benefits of CGMS

Participants across all groups recognized numerous benefits of using CGMS for diabetes management. These advantages included medical, social, and emotional benefits, its role as an educational tool, and notably, an overall enhancement in quality of life, particularly among T1DM patients.

#### Medical benefits

CGMS was highly praised for its real-time blood glucose monitoring, offering a more comprehensive view of glucose trends compared to traditional finger-prick testing. Participants emphasized the convenience and effectiveness of CGMS in enabling proactive lifestyle and insulin dosage adjustments, leading to improved glycemic control. For adolescents, parents highlighted the significant medical benefits of CGMS in managing unpredictable blood glucose fluctuations caused by hormonal changes, physical activity, and dietary variations. The real-time trend data allowed for immediate insulin dosage adjustments, reducing reliance on finger-prick tests. This proactive management contributed to improved glycemic control, lower HbA1c levels, and better overall health, enhancing both confidence and diabetes management in adolescents.

#### Social benefits

The convenience of CGMS significantly improved patients’ social experiences by reducing the need for frequent glucometer checks. Adolescents particularly highlighted the sense of freedom and carefree living that CGMS afforded them, enabling them to participate in social events without the constant interruptions of glucose monitoring. This newfound autonomy created a sense of normalcy in their social interactions, while caregivers appreciated the reduced burden of constant supervision and monitoring.

#### Emotional benefits

CGMS played a crucial role in reducing the emotional distress associated with frequent finger pricking, particularly among adolescents with T1DM, helping them feel more at ease and confident in their daily lives. It also alleviated discomfort, fears, and anxiety about managing blood sugar levels and provided caregivers reassurance, enhancing overall emotional well-being.

#### Improved quality of life

Participants unanimously agreed that CGMS technology significantly improved their overall quality of life by providing peace of mind and better sleep quality. The device empowered them to regain control over their diabetes management, fostering a strong sense of well-being and normalcy in their daily lives. The painless nature of the device and its role in alleviating concerns related to nocturnal glucose fluctuations were highlighted as tangible benefits, particularly among caregivers, who reported more restful nights as a result.

#### Patient educational tool

CGMS emerged as an essential educational tool, providing patients with a comprehensive understanding of their diabetes and empowering them to take proactive control of their management. Adolescents with T1DM particularly valued the independence it offered, enabling them to monitor their glucose levels, understand the impact of meals, exercise, and stressors, and make informed decisions with confidence. For caregivers, CGMS reduces the burden of constant supervision by providing real-time visibility into their adolescent’s diabetes status and timely alerts, minimizing the risk of hypoglycemic events. Additionally, CGMS data was invaluable for healthcare professionals, facilitating evidence-based adjustments to treatment plans and promoting more effective, personalized diabetes care.

### Perceived barriers to using CGMS

Patients and caregivers identified several barriers to adopting CGMS for diabetes management. The primary concern was the substantial financial burden associated with the device and frequent sensor replacements. Other significant barriers included device-related issues, limited accessibility and support, psychosocial challenges, and skin irritation.

#### Substantial financial impact

Participants consistently expressed concerns about the high costs of CGMS devices and the frequent need for sensor replacements due to their limited lifespan. The absence of insurance coverage, government subsidies, or financial support posed significant barriers to access, with some patients forced to discontinue CGMS use despite recognizing its benefits. Adolescents and their caregivers were particularly apprehensive about the long-term affordability of CGMS, especially as adolescents’ transition to adulthood and face financial challenges without government support. A notable finding was that some patients could only use CGMS intermittently due to financial constraints, highlighting the significant burden of maintaining consistent usage.

#### Device-related issues

Device malfunctions and sensor-related issues were frequently reported by participants, particularly sensor failures and dislodgement. Many described instances where sensors displayed ‘data not available’ or experienced data loss before reaching their intended lifespan, especially during the second week of use. Adolescents faced unique challenges, as sensor dislodgement often occurred during physical activities, such as sports, excessive sweating, or contact with their surroundings. This limitation sometimes restricted their participation in contact sports and other activities, impacting their daily lives. Despite these challenges, for the few participants whose CGMS devices had alarm functions, the alerts were generally considered unobtrusive and not a major concern.

#### Limited accessibility and support

Accessing CGMS devices was a significant challenge due to limited market availability. Participants often had to visit multiple pharmacies, resulting in occasional stock delays and extended waiting times. Patients and caregivers specifically noted difficulties in accessing the latest CGMS versions, which offer advanced features and greater convenience. Additionally, participants highlighted the lack of technical support and peer support groups as barriers to effective CGMS use. Some doctors were also perceived as lacking the necessary training to interpret CGMS data for medication adjustments.

#### Psychosocial issues

Social stigma and public perception were noted as significant barriers to CGMS adoption, particularly among adolescents with T1DM. Many adolescents shared feelings of self-consciousness and discomfort due to stares from peers or the public, stemming from limited awareness and acceptance of CGMS in Malaysia. For some, the fear of appearing ‘different’ or being perceived as ‘not normal’ led to hesitancy or avoidance of CGMS use. Additionally, participants and caregivers expressed stress and anxiety when replacing sensors independently, fearing device malfunctions and the lack of immediate technical support.

#### Skin irritation

A subset of participants experienced skin irritation at the sensor site, which limited their ability to use CGMS continuously. This issue created an additional barrier to consistent CGMS use, as users needed to allow their skin to heal between sensor placements.

### Expectations for CGMS adoption

#### Government advocacy and accessibility

Participants expressed the need for increased advocacy from government bodies to improve the accessibility of CGMS devices. They emphasized the importance of government initiatives aimed at subsidizing or providing CGMS devices at reduced costs, particularly for marginalized groups such as the lower-income category, senior citizens, and individuals at high risk of diabetes-related complications.

#### Expanded availability and support

Participants emphasized the need to improve the availability of CGMS in Malaysia and to establish comprehensive support programs for those adopting CGMS technology. They expressed interest in newer CGMS versions with advanced features and highlighted the importance of training healthcare professionals to ensure the optimal use of CGMS devices for effective diabetes management.

## Discussion

The focus group discussions revealed key insights into the experiences of individuals with diabetes and their caregivers using CGMS. Participants highlighted CGMS as a transformative tool that provides real-time data, serves as an educational resource, and improves glycemic control. They noted its positive impact on their diabetes management routines, reducing anxiety, and enhancing quality of life. These findings align with prior research by Lawton et al. (11), Peyyety et al. (12), Hilliard et al. (13), and an HTA conducted in Canada, which similarly highlighted the medical, emotional, and social benefits of CGMS (11-14). For instance, the Canadian HTA reported that participants valued CGMS for tracking blood glucose trends rather than relying on single data points from finger-prick tests, facilitating better insulin dosing and control (14). Both patients and caregivers appreciated the social freedom CGMS provided by enabling discreet diabetes management and promoting greater independence and personal control (14). Adolescents and caregivers in this study also emphasized the motivational aspect of visualizing glucose trends, which supported confidence and self-management in diabetes care. These findings are consistent with evidence showing that CGMS improves daily functioning, work performance, and interpersonal relationships, particularly for individuals with T1DM (15;16). While Lawton et al. (11) focused on T1DM patients and caregivers in the UK, and Peyyety et al. (12) and Hilliard et al. (13) examined youth with T2DM and young children with T1DM in the United States, respectively, this study sheds light on unique challenges within the Malaysian context, including financial barriers, limited public awareness, and social stigma, which were particularly pronounced among adolescents.

Despite the recognized benefits of CGMS, participants in this study also identified significant barriers to its use. Chief among these was the high cost of devices and the frequent need for sensor replacements, further compounded by the lack of insurance coverage and government subsidies. These financial challenges were particularly burdensome for lower-income groups and adolescents transitioning to adulthood. Research has demonstrated that subsidizing CGMS costs lead to increased uptake, improved adherence, and better glycemic outcomes for both T1DM and T2DM patients (17). Addressing these disparities is not only a healthcare priority but an ethical imperative. Expanding access to CGMS through targeted policies, such as government subsidies, insurance integration, or affordable payment plans, is critical to overcoming these barriers as CGMS could reduce costs for patients and yield long-term savings by preventing diabetes-related complications. Advocacy groups and healthcare policymakers play a vital role in driving these reforms, which could reduce long-term healthcare costs by preventing diabetes-related complications.

In addition to financial barriers, adolescents in this study highlighted specific challenges, such as the emotional impact of social stigma and difficulties with CGMS use during physical activities. Similar concerns were reported by Peyyety et al. (12), where stigma and device visibility were major barriers to CGMS uptake among youth with T2DM in the United States (12). Earlier, Messer et al. (15) also highlighted heightened concerns about device visibility and its impact on body image, which were observed not only among youth but also in adults with diabetes (15). In the Malaysian context, the social stigma surrounding CGMS use may stem from limited public awareness and understanding of diabetes management, leaving adolescents feeling self-conscious and “different” in social settings. Caregivers, particularly parents, emphasized the need for stronger diabetes education and community support programs to address these challenges. Public awareness campaigns and school-based diabetes education programs can play a vital role in reducing misconceptions and fostering a more inclusive environment for adolescents (18). By educating peers and educators, school-based programs can normalize CGMS use early on, improving understanding and acceptance. Additionally, peer support groups and group discussions can provide adolescents with platforms to share their experiences, reduce isolation, and build confidence in using CGMS (19). Collaborative efforts involving healthcare providers, educators, and advocacy groups are essential to creating a supportive ecosystem that encourages CGMS adoption and empowers adolescents to manage their diabetes confidently in socially challenging settings.

Technical limitations, including premature sensor failure, dislodgement, and data loss, were also recurring concerns in this study, particularly among adolescents whose active lifestyles and engagement in sports exacerbated sensor-related issues. Participants in the focus group discussions reported sensor dislodgement during activities such as sports and swimming, challenges further compounded by Malaysia’s hot and humid climate, which weakens adhesive stickiness. These findings align with Karakus et al. (20), where parents expressed concerns about reduced sensor tape adhesion due to increased sweating and swimming in summer, leading many to use extra adhesives to protect CGMS from water, impacts, and dislodgement (20). However, this solution often caused skin irritation, itching, and discomfort from prolonged adhesive use (20). Further supporting this, Messer et al. (15) highlighted concerns among individuals with T1DM regarding sensor dislodgement during everyday activities such as exercise or sports, mirroring the experiences shared by adolescents in this study (15). Similarly, Pickup et al. (21) noted adhesion problems, premature sensor failure, and incidents of sensors catching on clothing, with failure often occurring before the expected lifetime (21). These technical stressors were also reported by Hilliard et al. (13) among young children with T1DM, particularly those in active populations (13). This study further highlights the emotional burden of sensor replacement and the lack of comprehensive technical support in the Malaysian setting. Participants noted that while alarms and alerts were generally non-intrusive, the frustration of managing device malfunctions and unreliable sensors added significantly to their burden. Addressing these limitations requires manufacturers to improve sensor durability and reliability to better meet the needs of active users such as adolescents. Additionally, participants emphasized the lack of technical support and peer support networks, which limited their ability to troubleshoot device issues independently. Comparisons with findings from Lawton et al. (11) suggest that structured education programs and peer support groups could effectively address these challenges, empowering both patients and caregivers to maximize CGMS use (11). Future efforts to improve CGMS adoption should focus on enhancing technical support systems, strengthening peer networks, and raising awareness of advanced CGMS versions that address durability and reliability concerns. While skin irritation was less prevalent in this study, it remains an issue highlighted in previous research and warrants continued attention (22-24).

In light of these challenges, participants maintained a positive outlook on CGMS, recognizing its role in empowering diabetes management and improving overall well-being. Many expressed a willingness to recommend CGMS to others provided financial barriers, such as government subsidies, insurance coverage, and affordable payment options, are addressed. Participants were also interested in newer CGMS versions with advanced features, including improved alarms and better connectivity. Improving access and usability in Malaysia requires a coordinated approach, including policy-driven financial support, partnerships with manufacturers, public education initiatives, and enhanced training for healthcare providers to optimize CGMS use.

While this study provides valuable insights into CGMS use among Malaysian diabetes patients and caregivers, the relatively small sample (N = 30) limits generalizability. Although data saturation was achieved, the participants might not fully represent the broader population of CGMS users in Malaysia, potentially introducing sampling bias. Recruitment predominantly involved urban participants from Kuala Lumpur and Putrajaya, where access to CGMS and healthcare facilities is better, potentially underrepresenting rural populations who face greater barriers to healthcare. Although participants included various socioeconomic backgrounds, rural and underserved communities may have been underrepresented. This limitation likely skews findings towards perspectives of urban populations with better financial means and healthcare access. Consequently, the conclusions may be less applicable to rural or disadvantaged populations, where affordability and accessibility barriers are more pronounced. Future studies should include a larger, more diverse sample to capture the experiences of patients across different geographic, economic, and cultural settings in Malaysia. Additionally, social desirability bias could have influenced responses, and findings may reflect specific geographic and healthcare contexts rather than long-term trends.

To advance CGMS adoption in Malaysia, addressing financial barriers through actionable initiatives is essential. Implementing a tiered subsidy framework could ensure equitable access by aligning affordability with patients’ income levels, similar to models used for other essential medical devices. Bulk procurement agreements between the Ministry of Health and CGMS manufacturers could be leveraged in device price negotiations, while collaborating with insurers to include CGMS as a reimbursable device within healthcare benefits could further alleviate financial burdens, particularly for insulin-requiring T1DM patients. Public awareness campaigns targeting adolescents and rural communities, supported by community health centers and social media, are crucial for reducing stigma and increasing CGMS acceptance. Patient support groups, both in-person and virtual, can provide platforms for sharing experiences, addressing misconceptions, and offering emotional support. Collaboration between healthcare providers and manufacturers to enhance technical support and training for personalized medication adjustments is equally important. By addressing these barriers, stakeholders can improve CGMS accessibility, usability, and acceptance, ultimately enhancing diabetes management in Malaysia.

The findings from this qualitative study were integrated into the HTA alongside systematic reviews and a local economic evaluation. Patient insights provided critical real-world perspectives that complemented quantitative data from clinical trials and economic models. Feedback on CGMS’s role in improving glycemic control, reducing hypoglycemic events, and enhancing quality of life informed the interpretation of clinical effectiveness data. Additionally, patients’ concerns about financial barriers and device limitations highlighted key contextual factors influencing the economic evaluation and policy recommendations. This approach ensured a balanced, holistic assessment of CGMS, aligning healthcare decisions with patient needs. While CGMS demonstrated clear clinical benefits, the local economic evaluation found it was not cost-effective at the current price compared to self-monitoring of blood glucose (SMBG). Patient feedback emphasized CGMS’s perceived value in improving quality of life, empowering diabetes management, and reducing caregiver burden, which is not always captured in traditional cost-effectiveness analyses. Participants’ strong endorsements, particularly for adolescents managing glucose fluctuations, reinforced CGMS’s long-term benefits beyond direct economic outcomes. This intersection between patient insights and economic evaluation strengthened policy implications, leading to recommendations for targeted subsidies and improved financial access, particularly for insulin-requiring T1DM patients. A deliberative process with stakeholders, including policymakers, HTA experts, and endocrinologists, resulted in recommendations to offer CGMS for insulin-requiring T1DM patients at risk of severe hypoglycemic events. Addressing financial barriers, improving accessibility, and providing support are essential to fully leverage CGMS in diabetes management. The HTA report referenced in this work will be published online on the MaHTAS website in English.

Patient and public involvement (PPI) in HTA has been essential in guiding decisions regarding CGMS in Malaysia. Incorporating patient perspectives alongside systematic reviews and economic evaluations highlights how PPI enhances HTA’s relevance and depth. Insights from focus group discussions provided real-world evidence of CGMS’s benefits and challenges, including financial barriers, technical limitations, and social stigma. These contributions shaped HTA recommendations, ensuring they reflect end-users lived experiences and needs. The integration of patient insights in this study offers a replicable model for future HTA initiatives. Engaging patients and caregivers early in the assessment process allows HTA teams to capture perspectives that complement clinical and economic evaluations. This participatory approach ensures evidence-based and patient-centered healthcare decisions. Such a model can be scaled to evaluate other emerging health technologies across diverse contexts, promoting more equitable healthcare policies. The success of earlier PPI efforts in Malaysia, such as the HTAs on pre-dialysis education programs for advanced chronic kidney disease patients, prostate cancer screening, and computerized cognitive behavioral therapy for depression, demonstrates the value of integrating patient perspectives in shaping holistic recommendations (25-26). This approach aligns healthcare decisions with patient priorities, promoting tailored and effective solutions. By integrating patient insights with clinical and economic evidence, decision-makers gain a comprehensive understanding of CGMS, enabling more equitable and informed healthcare policies. Continued patient engagement in HTA processes is essential for improving technology adoption, enhancing health outcomes, and ensuring inclusive healthcare solutions in Malaysia and beyond.

## Conclusion

Focus group insights highlight the transformative impact of CGMS in diabetes management, providing real-time data, reducing the burden of glucose monitoring, and enhancing quality of life. Despite challenges like high costs and technical issues, the integration of patient perspectives as a vital voice in HTA has been instrumental in shaping policy recommendations, including prioritizing CGMS for high-risk T1DM patients and exploring subsidy frameworks to improve affordability. These patient insights, combined with evidence from systematic reviews and economic evaluations, ensure that healthcare decisions are informed by the lived experiences of those directly impacted. Moving forward, addressing financial barriers, enhancing technical support, strengthening patient support networks, and training healthcare providers are critical steps to fully realize CGMS’s potential in improving outcomes for individuals living with diabetes. By ensuring that patient voices remain central to HTA and strengthening partnerships across healthcare systems, the perspectives of patients will continue to inform decision-making on CGMS utilization and advance patient-centered healthcare policies in Malaysia.

## Supporting information

Mohamad et al. supplementary material Mohamad et al. supplementary material

## Supplementary material

The supplementary material for this article can be found at http://doi.org/10.1017/S0266462325000078.
