# Supplementary material for: Patient perspectives on continuous glucose monitoring system (CGMS) for diabetes in Malaysia: a vital voice in health technology assessment (HTA) informing decision-making
Source: Int J Technol Assess Health Care. 2025 Feb 6;41(1):e13. doi: 10.1017/S0266462325000078 (PMC11894388; doi:10.1017/S0266462325000078)
Supplement: Mohamad et al. supplementary material [file S0266462325000078sup001.pdf]

Appendix 1:

Patients' and Caregivers' Statements on Experiences with Continuous Glucose Monitoring System (CGMS)

Perceived Benefits of CGMS

**Medical Benefits**	*"With CGMS, it has a graph to view our blood sugar readings, we can see the trends, whether it's going up or down. We can tell when we eat, we can check, oh, it can show how much our blood sugar level is. So, we can control our diet."* (Adult with T1DM)<br><br>*"Also, this CGMS shows trends with arrows pointing up and down, so we can anticipate our next reading. It's indicative. We can know what our next blood sugar reading prediction will be like, so better control."* (Adult with T1DM)<br><br>*"Far, far different. When I do the prick test, I have to get up, do the finger prick test, swab my finger. With CGMS, I just need to turn on my phone and check. It feels safer, and I can monitor at any time."* (Adolescent with T1DM)<br><br>*"Even better because you can see it directly with CGMS. Another thing is it gives notifications if it's too low or too high. We can feel it, but what if the glucometer is suddenly left behind? So, with CGMS, it will always be with us."* (Adolescent with T1DM)<br><br>*“If without CGMS, cannot prick fingers too many times, and then you don't know what you eat. So with CGMS, we know where we are and its better. We have better sugar control. With CGMS, he (adolescent) only pricked finger only two times with maximum a day. Morning and then before bed. With the CGMS at least we have insight. You know he will not in the dangerous situation especially before sleep.”* (Caregiver of adolescent with T1DM)
**Social Benefits**	*"Before this, I had to carry a glucometer, strips, and all that, so this is easier. Because before CGMS, I had to carry all this every time I go out with friends and it was bothersome to do glucose checking and everything while my friends asked to go here and there, because they don’t need to do any of this."* (Adolescent with T1DM)<br><br>*"It feels more convenient because there's no need to draw blood anymore. Until now, I had to draw blood, but now, while watching TikTok or whatever, I just look at the side and see my blood sugar level. It's like an advancement. So, I can engage in many activities with my friends."* (Adolescent with T1DM)

	“Easy and quick. If you do finger-prick, it takes time. Previously, she had to do the prick before eating, so it took time during break time, friends had to wait for her, even to buy food, and the break time was only half an hour. But with CGMS, it's faster; she can inject insulin quickly and eat with friends." (Caregiver of adolescent with T1DM)<br><br>*“After some time then they get used to the CGMS, because they see the reading, they also can have more control, they have more freedom”* (Caregiver of adolescent with T1DM)<br><br>*"I think when I want to eat out, for example, when going out with friends, if I want to eat, I will check my blood sugar level. I can check more frequently, anytime, with CGMS. I can know my blood sugar readings."* (Adult with T1DM)
**Emotional Benefits**	*“You always feel that you're not normal. You're not part of the others who are playing their game, but you're not part of it. And this thing (CGMS) brought this life back now I can play again. With them.”* (Adult with T1DM)<br><br>*"Before this, because I had to prick my finger so many times a day, I was extremely stressed. Now, with CGMS, I’m happy."* (Adolescent with T1DM)<br><br>*"Previously, he was very stressed. So, he was very tense, and his fingers even became stiff because of frequent pricking, so he wouldn't check. He was stressed about having to prick so many times. With CGMS, he is much, much happier."* (Caregiver of adolescent with T1DM)<br><br>*"CGMS reduced the stress and anxiety of my child because she couldn't bear to do the pricking anymore, and it was heart breaking to see her."* (Caregiver of adolescent with T1DM)<br><br>*"It's convenient, and I'm less worried. I feel safer. We can easily see the glucose readings by looking at the reading trends."* (Caregiver of adolescent with T1DM)
**Improved Quality of Life**	*“Night-time, its better now. Before going to bed, I check my blood sugar level first. So, I can make adjustments. I can sleep well now.”* (Adolescent with T1DM)<br><br>*“Before that, one year plus without CGMS, I was in the dark, I only sleep for two hours and check and see if everything is OK. Cannot have a good sleep. Now I can sleep with no problem. Everything is good now.”* (Caregiver of adolescent with T1DM)

	"From black before the time of CGMS to white. With this thing I can live. Yeah, I'm living with diabetes with this thing. Without it, I'm just surviving." (Adult with T1DM)<br><br>*"I think that we can all hear that what we are trying to emphasize here, because of this CGMS, it gives you peace of mind."* (Adult with T1DM)<br><br>*"CGM improves my life, it controls the disease, without it, disease controlled us."* (Adult with T2DM)
**Patient Educational Tool**	*"Yes, because with this thing (showing CGMS), I can really understand my diabetes. I can go out. I can do everything. I can go to the cinema without hesitating and survive the evening. It is really that important for me."* (Adult with T1DM)<br><br>*"Now with CGMS I can do everything myself; I can manage my own blood sugar, my food, my activities."* (Adolescent with T1DM)<br><br>*"At night, it's better. Before going to sleep, I check my blood sugar levels first. So, I can adjust it myself."* (Adolescent with T1DM)<br><br>*"With CGMS, it's supposed to be helping the doctors as well. We have our Endocrine doctor. Every time we want to adjust insulin or even in the hospital, the doctor also based on some basic information to calculate how much basal per hour and what not. That kind of things with the CGMS reading then the doctor can adjust better."* (Caregiver of adolescent with T1DM)
**Perceived Barriers to using CGMS**	
**Substantial Financial Impact**	*"The main thing is the price. It's just too expensive for me. The price for the device is already expensive, then I have to change the sensor which costed me another RM 200 to 300 (45 to 67USD) every 10-14 days, it is very expensive"* (Adult with T1DM)<br><br>*"At the moment, the problem is the cost. The cost for this device, and cost for sensor is very expensive. The only thing is I wish the price could get lower."* (Adult with T1DM)

	"So, for me, it's also difficult because this CGMS is expensive, the cost is high, so I don't know how it will be when I grow up, and my parents are no longer working, so how will I continue, how will I pay for it." (Adolescent with T1DM)<br><br>*"Currently, it's my parents who are covering the cost, but now I'm thinking a lot about how the expenses for CGMS will be in the future. My parents can afford it, but I still worry that it might be burdensome for them."* (Adolescent with T1DM)<br><br>*"Cost is a big issue, the device, the sensor, it's crazy and too much. I wish to have it long term if cost wasn't the issue."* (Caregiver of adolescent with T1DM)<br><br>*"But overseas, they all have insurance. In Malaysia, we don't have that. There's no room for people with diabetes to get insurance, even if they're willing to pay the premium. There's no subsidy from the government, no assistance for CGMS."* (Adult with T1DM)<br><br>*"For 3 months, I can still manage, but for the long term, I won't be able to afford it. Maybe I won't be able to use it continuously, once a month or every two months, I might be able to manage, but for continuous use, it's not affordable for me."* (Caregiver of adolescent with T1DM)
**Device-related Issues**	*"But usually, when it's about to reach the 2-week mark, I feel it becomes a bit 'faulty.' It keeps showing 'glucose level not available' suddenly, and during the second week, it's consistently faulty before it should be, unable to detect, sensor not available."* (Adolescent with Type 1 diabetes)<br><br>*"Then, if I feel like 'data not available' is taking too long, I'll refresh it, then wait for notifications; sometimes it's okay, and sometimes I have to change the sensor. This often happens during the second week, around day 9-10, and it consistently occurs."* (Adolescent with T1DM)<br><br>*"I had sensors that didn't start at all. I had sensor to stopped after 5 to 6 days. And almost none of them lasted 2 weeks. Yesterday I've changed it to a new one because that one was 10 days. And it will tell you to just change the sensor. Even though it's not yet 14 days"* (Adult with Type 1 diabetes)<br><br>*"Yes, it happened few times, it got dislodged because someone bumped into me. So, the sensor was wasted, and I had to replace it with a new one."* (Adolescent with T1DM)

	"Earlier this year, I had indeed stopped playing soccer because I want to avoid getting sensor dislodged." (Adolescent with T1DM)<br><br>*"Contact sports have to be reduced because CGMS can get detached."* (Caregiver of adolescent with T1DM)
**Limited Accessibility and Support**	*"Basically, I don't know where to get it. Then, I travel all the way from my house and I want to get it, this CGMS is not available."* (Adult with T1DM)<br><br>*"Very stressful for us when we had to set up the CGMS and when something is wrong, it's hard to get immediate help."* (Caregiver of adolescent with T1DM)<br><br>*"Sometimes, we need a support group for CGMS users to exchange our issues so that we can make it easier to find out where to get CGMS, learn from the experiences of those who have been using it for a long time. But there isn't one."* (Adult with T1DM)<br><br>*"One thing here in Malaysia we have only old version while people overseas have other better versions with better design and functions."* (Caregiver of adolescent with T1DM)
**Psychosocial Issues**	*"All guys are my age and they are normal and when they are asking what is that, giving me pity eyes. So that's another reason why I didn't want to use it."* (Adult with T1DM)<br><br>*"You know, socially in Malaysia is not widely accepted yet. Everybody start questioning what is it? What is it? Because many people don't know and I don't want every one of them, looking at me like that"* (Adult with T1DM)<br><br>*"Many of my friends don't know about my illness. So, some people find me strange."* (Adolescent with T1DM)<br><br>*"I also wonder why people find it strange. At the same time, when my child uses CGMS and we must do checks, others look at us, they find it strange, like, 'Why is this child doing that?"* (Caregiver of adolescent with T1DM)
**Skin Irritation**	*"CGM cause skin problem, like the skin becomes irritable. Had skin issue when long term and no place to put anymore when the skin irritation problem because you repeatedly use the skin. So, we cannot use continuously. We have to let the skin rest for few weeks."* (Caregiver of adolescent with T1DM)

	"I found my skin to be irritable and itchy around the sensor area" (Adolescent with T1DM)
**Expectations for CGMS adoption:**	
**Government Advocacy and Accessibility**	*"If it's free, I would definitely like it. Even if it's not free but subsidized, it would lessen the cost burden. If more of these can be made available in Malaysia, more people can use it. Because many families can't afford to own CGMS. Parents want the best for their young children who have been diagnosed, but they can't afford to get CGMS."* – (Caregiver of adolescent with Type 1 diabetes)<br><br>*"So you have to decide that that some categories should get free, you know, some categories of patients. It should be given to selected group of patients because it saves lives."* (Adult with Type 2 diabetes)
**Expanded Availability and Support**	*"We hope for better access and better ones to be available in Malaysia. Right now the process takes too much time and the newer versions are not here."* (Adult with T1DM)<br><br>*"If we can get the new, better version of CGMS in Malaysia, that would be the best because, for now, there isn't any."* (Adult with T1DM)
